# Supplementary material for: Assessment of the Contribution of a Thermodynamic and Mechanical Destabilization of Myosin-Binding Protein C Domain C2 to the Pathomechanism of Hypertrophic Cardiomyopathy-Causing Double Mutation MYBPC3Δ25bp/D389V
Source: Int J Mol Sci. 2021 Nov 4;22(21):11949. doi: 10.3390/ijms222111949 (PMC8584774; doi:10.3390/ijms222111949)
Supplement: Supplementary file 1 [file ijms-22-11949-s001.zip › ijms-1437366-supplementary.pdf]

Supplementary Information for

Assessment of the Contribution of a Thermodynamic and Mechanical Destabilization of Myosin–Binding Protein C Domain C2 to the Pathomechanism of Hypertrophic Cardiomyopathy–causing Double Mutation *MYBPC3*$^{\Delta 25bp/D389V}$

Frederic V. Schwäbe [1], Emanuel K. Peter[1], Manuel H. Taft[1] and Dietmar J. Manstein[1,2,*]

[1] Institute for Biophysical Chemistry, Hannover Medical School, Fritz Hartmann Centre for Medical Research Carl Neuberg Str. 1, D-30625 Hannover, Germany

[2] Division for Structural Biochemistry, Hannover Medical School, Carl Neuberg Str. 1, D-30625 Hannover, Germany

* Correspondence: manstein.dietmar@mh-hannover.de

Supplementary Figures

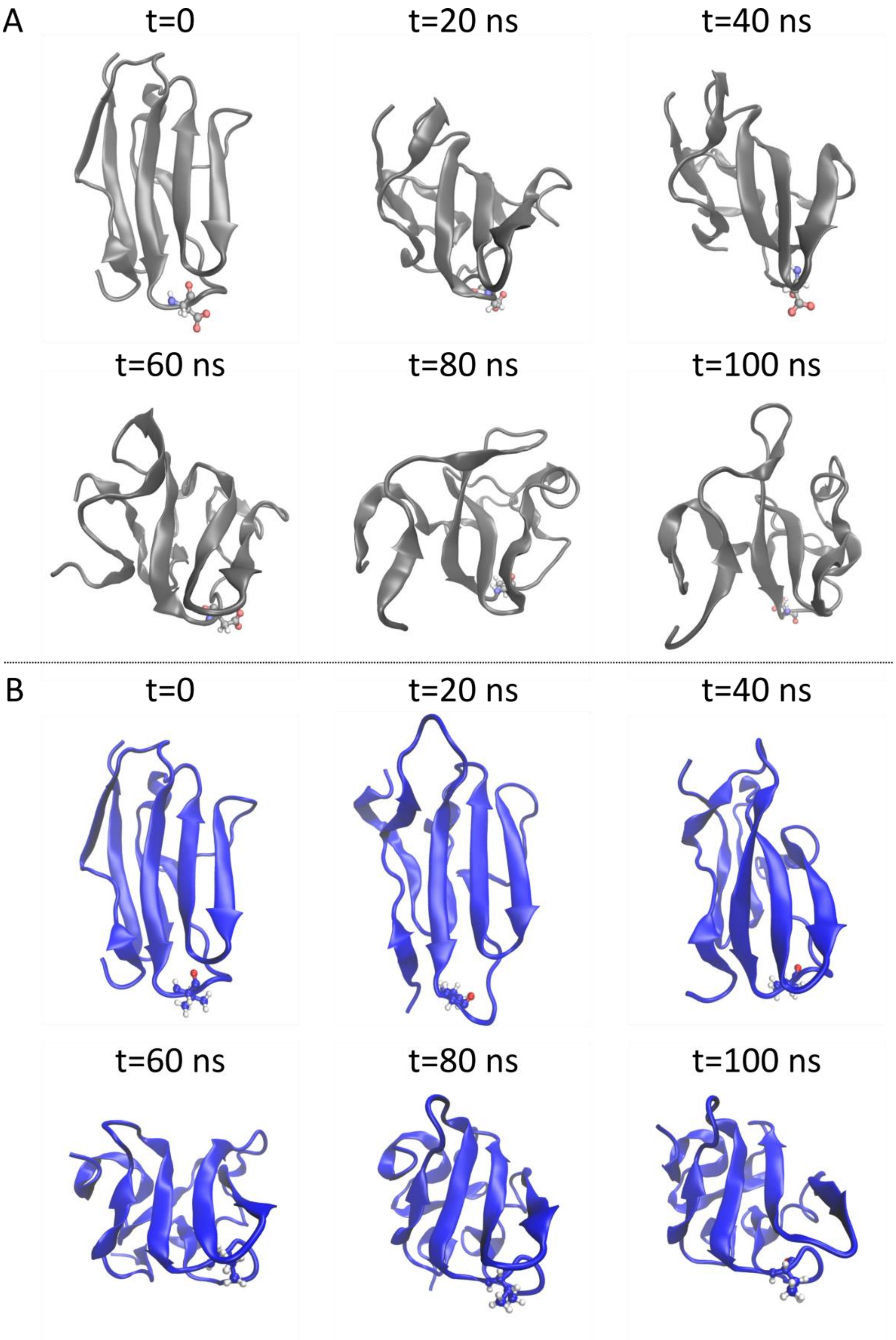

Figure S1. Sample conformers obtained from a 100 ns CORE-MD II simulation of MyBPC C2 wt (A) and D389V (B) domain. Wt structures are shown in grey and D389V in blue. Asp389 and Val389 are shown as CPK representations. Structures of time points from 0-100 ns are shown in increments of 20 ns.

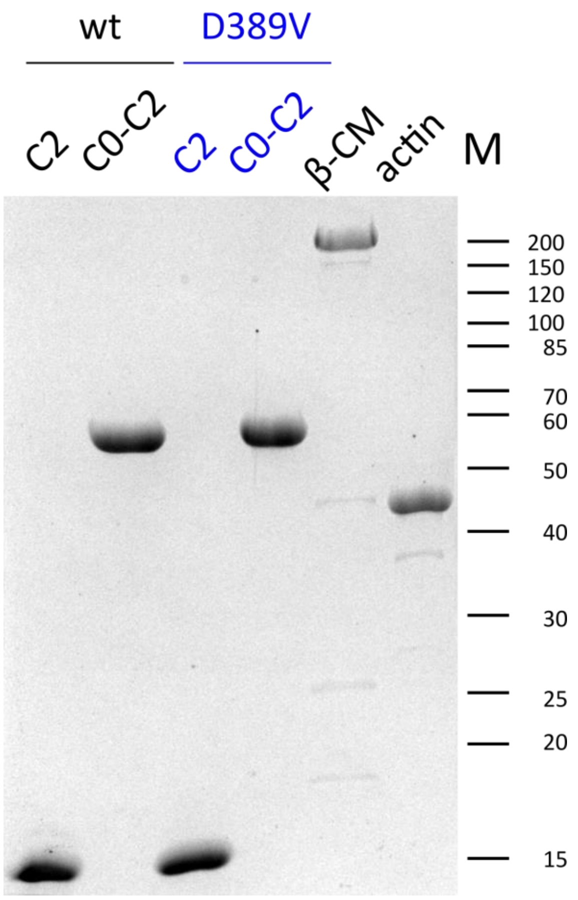

Figure S2. SDS gel of purified proteins used in the biochemical experiments. C2: MyBPC C2, C0–C2: MyBPC C0–C2 wt, β-CM: full-length β cardiac myosin, actin: α-cardiac actin. M: Molecular weight corresponding to the protein bands is indicated corresponding to PageRuler Unstained Protein Ladder. *Homo sapiens* MyBPC constructs were purified from *E. coli* and myosin and actin were purified from *Sus scrofa* native cardiac tissue.

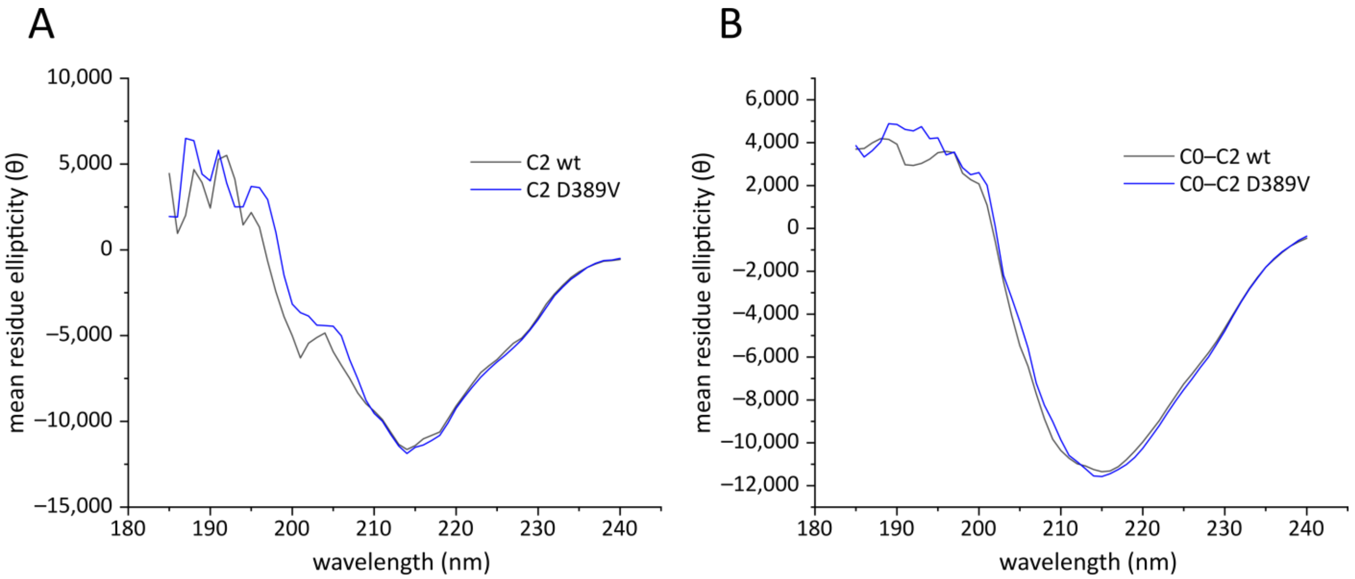

Figure S3. Circular dichroism spectra of MyBPC C2 and C0–C2 domains. Data were processed using DichroWeb server. Mean residue ellipticity is plotted against the wavelength for (A) C2 wt and D389V and (B) C0–C2 wt and D389V.

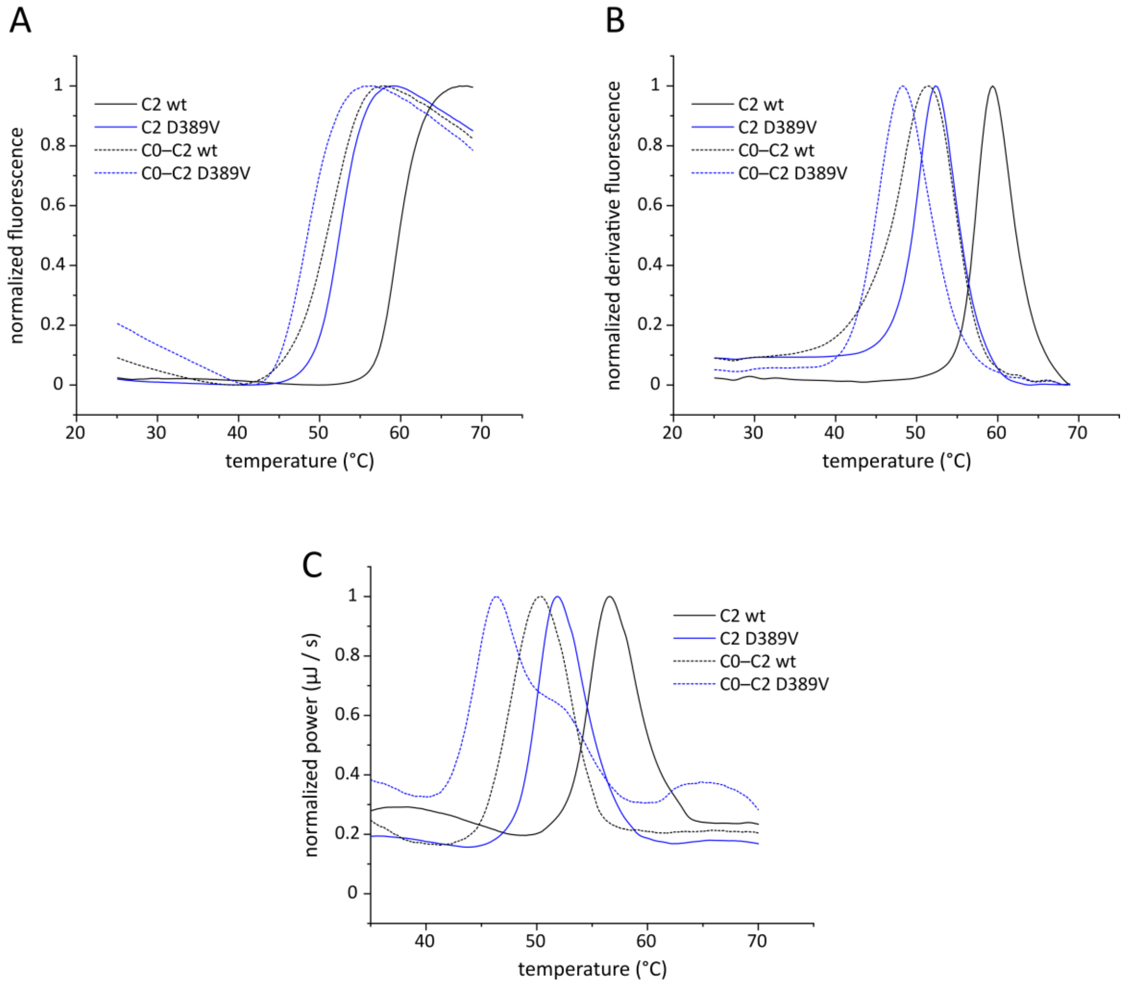

Figure S4. Analysis of thermal stability of N-terminal MyBPC constructs. C2 is shown as solid lines, C0–C2 as dashed lines; wt protein is displayed as black and D389V as blue. Representative (A) normalized and (B) normalized derivative fluorescence traces resulting from a thermal shift assay (TSA). Protein melting temperature (T_m) was determined as the temperature value at the maximum of the first derivative. (C) Representative normalized power traces of a differential scanning calorimetry experiment (DSC). T_m corresponds to the maximum of each trace.

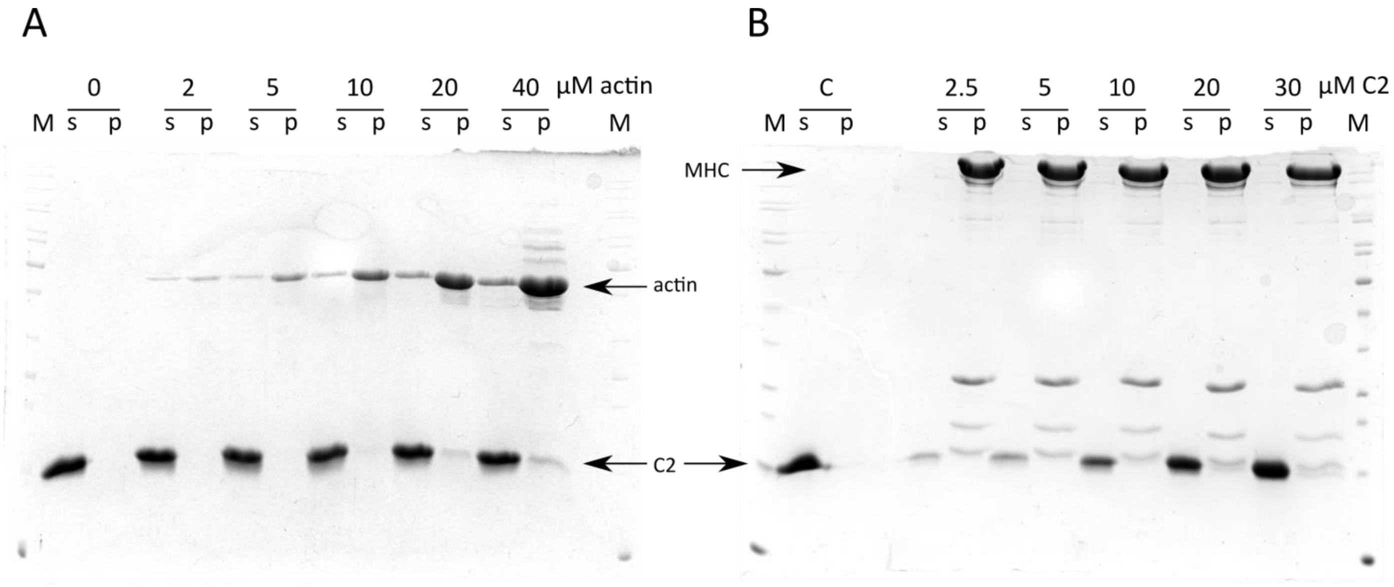

Figure S5. Cosedimentation assay of MyBPC C2 with F-actin and β-cardiac myosin. (A) Representative SDS gel of a high-speed co-sedimentation assay with 0 – 40 μM actin and 30 μM MyBPC C2. M: PageRuler Unstained Protein Ladder, 0 – 40: total concentration of actin in μM, s: supernatant, p: pellet. Densitometric analysis yielded <2% MyBPC C2 in the pellet fraction with the highest actin concentration (B) Representative SDS gel of a high-speed co-sedimentation assay with 2.5 μM β-cardiac myosin and 2.5-30 μM MyBPC C2. Densitometric analysis yielded <2% MyBPC C2 in the pellet fraction with the highest C2 concentration. M: PageRuler Unstained Protein Ladder, 0-30: total concentration of C2 in μM, s: supernatant, p: pellet, MHC: myosin heavy chain, s: supernatant, p: pellet, C: 30 μM C2 without myosin.

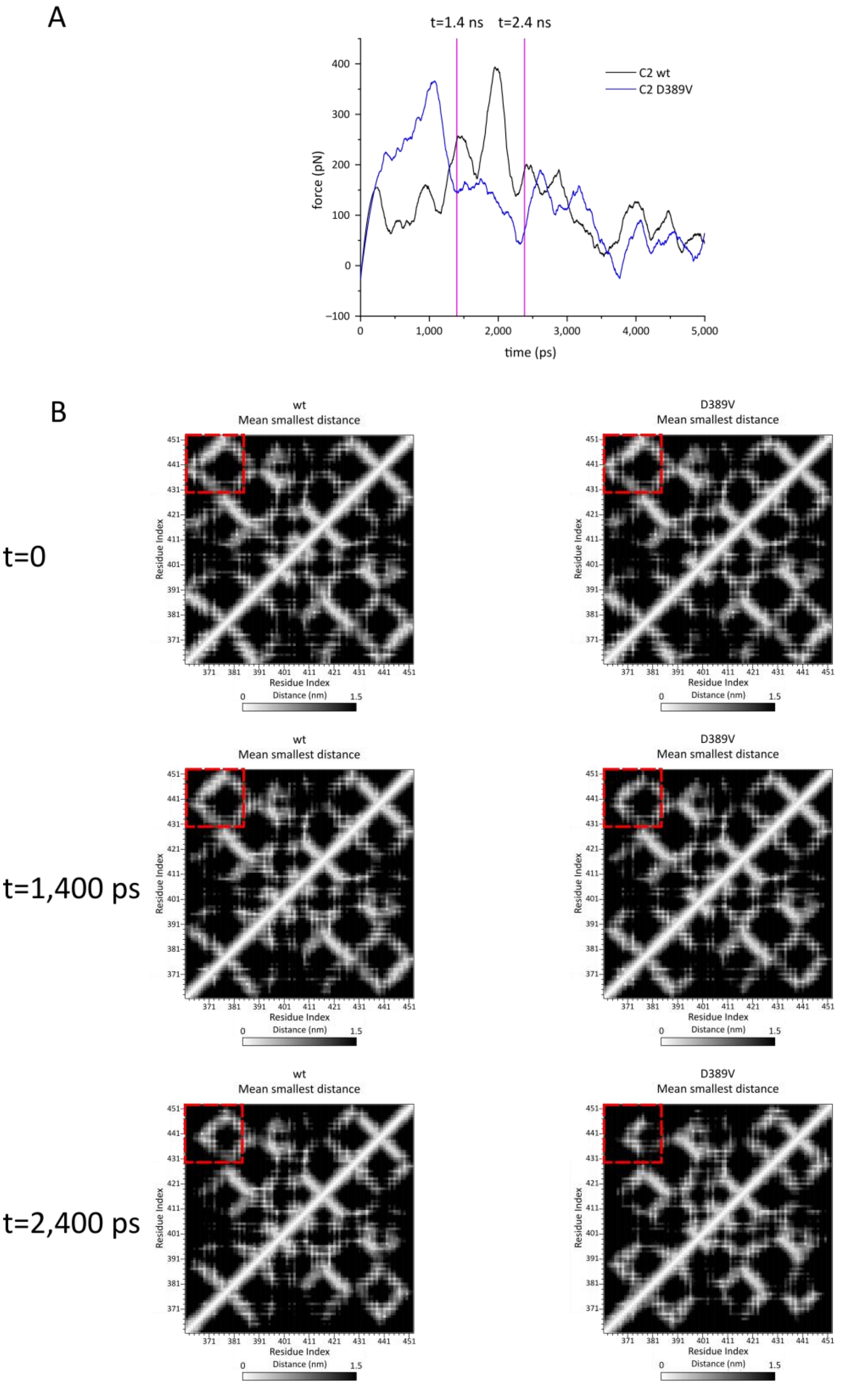

Figure S6. Constant velocity steered molecular dynamics (cvSMD) simulation of MyBPC C2 domain unfolding. The SMD atom was pulled at a velocity of 0.01 Å/ps and the spring constant was set to 7 kcal/mol/Å. (A) Sample traces for an unfolding simulation of each wt and D389V MyBPC C2 domain. The force acting on the SMD atom is plotted against the time of the simulation. Data were smoothened using Savitzky-Golay filter to compensate for the noise created by the spring acting on the fixed atom. (B) Mean smallest distance maps of time points 0, 1400 and 2400 ps in the unfolding experiments displayed in (A). The time-averaged minimum distance of each residue to every other residue is plotted in heat maps with white indicating the smallest and black the largest distance ranging from 0–1.5 nm. This map reveals differences in the global conformational space of C2 wt and D389V. Dashed boxes indicate an N-to-C-terminal β-sheet contact of C2 wt that is destabilized in C2 D389V.
